# Effects of the COVID‐19 pandemic in a preexisting longitudinal study of patients with recently diagnosed bipolar disorder: Indications for increases in manic symptoms

**DOI:** 10.1002/brb3.2326

**Published:** 2021-09-23

**Authors:** Manja Koenders, Rahele Mesbah, Annet Spijker, Elvira Boere, Max de Leeuw, Bert van Hemert, Erik Giltay

**Affiliations:** ^1^ Department of Psychiatry Leiden University Medical Centre Leiden The Netherlands; ^2^ Faculty of Social Sciences Institute of Psychology Leiden University Leiden The Netherlands; ^3^ Department of Mood Disorders Mental Health Care PsyQ Kralingen Rotterdam The Netherlands; ^4^ Parnassia Groep Den Haag The Netherlands; ^5^ Mental Health Care Rivierduinen Bipolar Disorder Outpatient Clinic Leiden The Netherlands

**Keywords:** bipolar disorder, COVID‐19, depression, loneliness, mania, stress

## Abstract

**Background:**

The coronavirus disease 2019 (COVID‐19) pandemic interfered in the daily lives of people and is assumed to adversely affect mental health. However, the effects on mood (in)stability of bipolar disorder (BD) patients and the comparison to pre‐COVID‐19 symptom severity levels are unknown.

**Method:**

Between April and September, 2020, symptoms and well‐being were assessed in the Bipolar Netherlands Cohort (BINCO) study of recently diagnosed patients with BD I and II. The questionnaire contained questions regarding manic and depressive symptoms (YMRS and ASRM, QIDS), worry (PSWQ), stress (PSS), loneliness, sleep, fear for COVID‐19, positive coping, and substance use. As manic, depressive and stress symptoms levels were assessed pre‐COVID‐19, their trajectories during the lockdown restrictions were estimated using mixed models.

**Results:**

Of the 70 invited BD patients, 36 (51%) responded at least once (mean age of 36.7 years, 54% female, and 31% BD type 1) to the COVID‐19 assessments. There was a significant increase (X^2^ = 17.06; *p* = .004) in (hypo)manic symptoms from baseline during the first COVID‐19 wave, with a decrease thereafter. Fear of COVID‐19 (X^2^ = 18.01; *p* = .003) and positive coping (X^2^ = 12.44; *p* = .03) were the highest at the start of the pandemic and decreased thereafter. Other scales including depression and stress symptoms did not vary significantly over time.

**Conclusion:**

We found a meaningful increase in manic symptomatology from pre‐COVID‐19 into the initial phases of the pandemic in BD patients. These symptoms decreased along with fear of COVID‐19 and positive coping during the following months when lockdown measures were eased.

## 1 INTRODUCTION

By the beginning of 2020, the coronavirus disease 2019 (COVID‐19) outbreak started to spread around the world. In order to contain the virus, drastic measures were needed that interfered in the daily lives of people. The threat of the disease itself and the disruptive daily‐life consequences of lockdown measures appear to have significant impact on mental well‐being. Previous research on the effects of quarantines (non‐COVID‐19 related) showed significant negative psychological impact on the general population (e.g., post‐traumatic stress, frustration, anger, fear, and boredom) (Brooks et al., 2020). Preliminary studies on the effects of COVID‐19 lockdowns reported adverse changes in sleep patterns and decreased quality of sleep (Cellini et al., 2020). Internet searches on topics such as boredom, loneliness, worry, and sadness increased drastically, suggesting that mental health is affected by the measures (Brodeur et al., 2020). Additionally, in an online survey among 1210 healthy respondents, 55% experienced a moderate to severe psychological impact, and 17% and 29% reported depressive and anxiety symptoms, respectively. Most respondents were worried about family members becoming infected (75%) and spent 20−24 h per day at home (85%). Women and those with physical symptoms and poorer health had higher anxiety and depression (Wang et al., 2020).

Because of these reported negative effects on the general population, several concerns have been raised regarding its effects on patients with psychiatric disorders (Yao et al., 2020). There are indications that people with higher symptom levels of depression, anxiety, and stress symptomatology suffer most from the lockdown measures (Cellini et al., 2020). People suffering from psychiatric disorder were found to be more severely impacted by the pandemic than healthy controls and reported (cross‐sectionally) more severe increases of stress‐related symptomatology like depressed mood, anxiety, and sleep problems (Hao et al., 2020).

Among people suffering from psychiatric disorders, a specifically vulnerable group to the effects of the COVID‐19 measures might be patients suffering from bipolar disorder (BD), especially because major life events are consistently identified as triggers for mood instability in BD (Lex et al., 2017). Obviously, a pandemic is not comparable to major life events such as the loss of a close relative, getting married, or moving house. Natural disasters might be comparable to only some extent, in which the current pandemic disrupts daily lives of almost all citizens. Although the influence of a pandemic on patients with BD is relatively unknown, a previous study on the effect of the Fukushima disaster already showed that a major life event could specifically exacerbate symptoms of BD, especially manic symptoms (and did not lead to increase in symptomatology in other psychiatric disorders) (Matsumoto et al., 2014). In case of the COVID‐19 pandemic, the lockdown measures seemed to interfere specifically with factors that are essential for bipolar mood stability, affecting social rhythm and sleep. This may induce a relapse into both depression and (hypo)mania (see reviews by Abreu & Braganca, 2015; Takaesu, 2018). A study into the effect of a natural disaster (earthquake) on bipolar and schizophrenic patients showed that both groups reported less social support and more avoidance (Horan et al., 2007). Recent cross‐sectional studies have shown that COVID‐19 pandemic was associated with a higher frequency of depressive episodes and alterations in biological rhythm in patients with BD (Carta et al., 2021; Karantonis et al., 2021; Van Rheenen et al., 2020).These studies found associations between lockdown measures and depression severity and alterations in biological rhythm (including impaired sleep, activity, and social rhythm) (Carta et al., 2021), with a potential increased effect on BD patients compared to unipolar patients and healthy controls (Van Rheenen et al. 2020). The later study was extended with verified BD diagnoses (*N* = 43) and the findings revealed relatively mild (mostly non‐significant) pandemic‐related depressive mood symptoms, which was ascribed to resilience (Karantonis et al., 2021). Finally, in an observational prospective study on affective disorders that included BD (*N* = 194), a low impact of COVID‐19 on mental health was found (Tundo et al., 2021), similar to our findings. In order to effectively weigh the impact of the COVID‐19 outbreak, comparison of pre‐COVID‐19 and post‐COVID‐19 severity levels is needed. Only few studies to date have been able to study the mental health impact of the COVID‐19 using such a design. One of those studies showed that patients with and without depressive, anxiety, or obsessive‐compulsive disorders experienced an adverse impact on their mental health from the COVID‐19 pandemic. Yet, those with the highest burden of mental illness tended to show no increase or even a slight symptom decrease (Pan et al., 2021). We are aware of only two studies on the impact of COVID‐19 in patients with BD that compared prepandemic symptom levels to post‐pandemic levels. In the study by Orhan et al. (2020), among older (over age 50) patients with BD comparable results with the study we previously mentioned were found: No worsening of symptom levels were observed among these patients. Actually, symptom levels significantly decreased among these patients. They did find that passive coping and loneliness were associated with symptom increase. The results of a 1‐month prospective study (Yocum et al., 2021) showed that BD patients were more affected by the lockdown restrictions with regard to life impact changes in biological and social rhythm, income and employment, and pandemic stress compared to healthy controls. Interestingly, the healthy control group showed an increase in depressive symptom severity during the pandemic when compared to prepandemic scores, whereas BD patients did not show a significant change in symptom severity.

The current study longitudinally investigates the effect of COVID‐19 measures in the Netherlands in an existing cohort of recently diagnosed and relatively young adults with BD, who were followed from the first months of the Dutch lockdown restrictions, into the period in which measures were temporarily eased. Of all participants, prepandemic data on symptom levels and perceived stress is available. The aim of the study is to investigate the mental health impact of the pandemic in bipolar disorder patients in terms of symptoms levels, loneliness, worry, stress, and specific COVID‐19‐related factors (e.g., fear of COVID‐19, coping). We additionally investigate whether specific factors are related to an increase in manic and depressed symptomatology.

## 2 MATERIALS AND METHODS

### 2.1 Participants

Patients from the Bipolar Netherlands Cohort (BINCO) study were enrolled in the current study. This is a Dutch cohort in which recently diagnosed (<1 year) bipolar I and II patients are included from different mental health outpatients clinics in the Netherlands. Clinical data such as mood status and received treatment are collected every half year; cognitive function, lifestyle factors, psychological characteristics, genetic, neuro‐imaging, endocrine, and immune status are assessed at baseline and after 1 year. Of the 70 patients that were enrolled in the study, 36 were willing to participate in the current substudy.The group included (*n* = 36) is compared with the group of subjects who did not participate (*n* = 34) in this substudy. There were no significant differences in age, gender, type of BD, mania (YMRS), and depression (QIDS) total scores between these two groups at baseline.

### 2.2 Procedure

This is an ecological add‐on study to BINCO which was approved by the medical ethical committee of the Leiden University Medical Centre (reference number NL51776.058.14, BINCO). We aimed to observe the incidence of mood change during the current COVID‐19 epidemic in the Netherlands. After verbal agreement to participate in the study, participants received the first online questionnaire. Because no face‐to‐face contacts were allowed during this period, participants signed informed consent in the online questionnaire. Subsequently, they received a repeated online questionnaire every month, with an additional telephone interview. In total, there were six repeated measurements (timepoint 1 [T1] to timepoint 6 [T6]), starting from April 2020 when the first lockdown in the Netherlands started, into October when the second corona wave started. During the summer (July and August), there was a break and there were no measurements. Figure S1 gives an overview of the timing of the measurements in relation to the COVID‐19 pandemic in the Netherlands, and its related lockdown measures. During the initial months of the pandemic, the strictest measures came into effect by the midst of March 2020, when schools, universities, day‐care, bars, restaurants, and other public places were closed. Most working people were only allowed to work from home, and clear restrictions were set on social gatherings inside and outside people's homes. Also, mental health care was mainly delivered through telephone‐ or video consultations. The Dutch measures were slightly less strict compared to other European countries, since people have been advised to stay at home, but were still allowed to go outside as long as 1.5 m (5 ft) social distance was maintained. Nevertheless, the measures had a significant impact on the daily lives of people.

The response rate was good for the majority of the follow‐up measurements, with a relatively low response on T5: T1: 92% (*N* = 33), T2: 83% (*N* = 30), T3: 83% (*N* = 30), T4: 67% (*N* = 24), T5: 33% (*N* = 12), T6: 78% (*N* = 28).

### 2.3 Materials

The Composite International Diagnostic Interview (CIDI) 2.1 Lifetime Dutch version (Section E, depression; Section F, mania) was used to confirm bipolar diagnosis in the BINCO sample at pre‐COVID‐19 baseline. The assessment of the CIDI was not part of the current COVID‐19 procedure.

The following measurements were repeatedly assessed in this substudy:

#### 2.3.1 Corona‐specific questionnaires

To assess Corona‐related information, we composed a brief questionnaire containing four subgroups of items: fear of COVID‐19 (six items; Cronbach's α = ·81), positive coping (six items; α = .79), sleep disturbance (three items, α = .60), and alcohol use and smoking (two items, α = .70). This questionnaire was adapted from another recent study into the effects of COVID‐19 on psychiatric patients (Pan et al., 2021). Answer categories were on a 5‐point Likert scale: 1 (completely disagree) to 5 (completely agree). A complete listing of the items is given in Table S1.

#### 2.3.2 Questionnaires on psychological well‐being

##### Depressed symptoms

The 16 items quick inventory of depressive symptomatology (QIDS‐SR) (Rush et al., 2003) was used to repeatedly assess symptoms of depression in the past 2 weeks. The QIDS‐RS questionnaire was previously completed at baseline (prepandemic) and during the six follow‐up measurements during the pandemic. The questionnaire covers the nine DSM 5 criteria of depression and has good internal consistency, also for the Dutch translation (Cronbach's alpha > .86) (Schulte‐van Maaren et al., 2013). Cronbach's alpha in the current study was .82.

##### Manic symptoms

In order to assess (hypo)manic symptoms both the clinician‐rated, 11‐item young mania rating scale (YMRS) (Young et al., 1978) and the 5‐item self‐report Altman self‐rating mania scale (ASRM) (Altman et al., 1997) were used.

The YMRS was assessed at baseline (prepandemic) and five times during the follow‐up measurement by brief telephone interviews. The items are scored based on the patient report and the clinical impression by the interviewer. The YMRS has good inter‐rater reliability (*r* = .93) (Young et al., 1978) which has been confirmed for the Dutch translation (Lukasiewicz et al., 2013). Internal consistency (Cronbach's alpha) in the current sample was .85.

The ASRM is a five‐item self‐report measure of current (hypo)manic symptoms (Altman et al., 1997). Total scores of ≥ 6 indicate a high probability of a manic or hypomanic state. The questionnaire has good internal consistency (Cronbach's alpha = .79). It has the ability to detect hypomania or mania with a sensitivity of .85 and a sensitivity of .87 (Altman et al., 1997). The Dutch version has not yet been validated.

##### Loneliness (DeJong Q)

For the assessment of loneliness, the 6‐item Jong loneliness scale‐short version (DeJong Q) was used. This questionnaire assesses social (e.g., number of relationships) and emotional loneliness (e.g., aspired relationships) on a 3‐point scale (no, more or less, yes), resulting in a minimum score of 0 and a maximum score of 12. The short version has good test‐retest reliability, ranging between *r* = 0.81 to *r* = 0.95 in different samples (De Jong Gierveld & Van Tilburg, 2010). Internal consistency of the scale in the current sample was 0.72.

##### Worry (PSWQ)

To assess changes in the reported amount of trait worry in the Penn State Worry questionnaire (PSWQ) (Meyer et al., 1990), an abbreviated 11‐item version was used (Antypa et al., 2017). The Dutch translation has good internal consistency in clinical samples (> .83) (Cronbach's alpha) (Kerkhof et al., 2000; van der Heiden et al., 2010). The questionnaire assesses pathological worry and its characteristics on a 5‐point Likert scale. Internal consistency of the Worry questionnaire in the current sample was .91 (Cronbach's alpha).

##### Perceived stress

The perceived stress scale (PSS) 10‐item short version (Cohen et al., 1983) was used to measure changes in the amount of stress patients subjectively experienced in the past 2 weeks. This questionnaire was completed at baseline (prepandemic) and during the six follow‐up measurements during the pandemic. The questionnaire measures the extent to which respondents consider their lives to be unpredictable, uncontrollable, and overloaded (e.g., in the last 2 weeks, how often have you felt nervous and "stressed"/been able to control your irritations, etc.) on a 5‐point Likert scale. The scale has good internal consistency (Cronbach's alpha = .82) (Roberti et al., 2006). In the current sample, the internal consistency of this questionnaire was .86 (Cronbach's alpha). The questionnaire has been validated in numerous languages, but the Dutch version has not yet been validated (Lee, 2012).

### 2.4 Statistical analyses

Baseline characteristics and sociodemographics were summarized as means (with standard deviations [SD]) for continues variables and as numbers for proportions for categorical variables. We considered a *p*‐value less than .05 statistically significant. We averaged assessments of the baseline and 1‐year assessments for the YMRS, QIDS, and PSS severity scores that took place in 2017 and 2019 to yield the pre‐COVID‐19 severity levels among the 36 participants.

We used exploratory factor analysis (EFA) with principal axis factoring and oblique rotations (i.e., Oblimin) to examine dimensionality of COVID‐19‐specific questionnaire. Four dimensions were determined based on the screeplot, evaluation of Eigenvalues (>1 indicates a distinct dimension), factor loadings, and conceptual plausibility. The four dimensions in the COVID‐19‐specific items were labeled as fear of COVID‐19, positive coping, sleep disturbance, and alcohol use and smoking. See Table S1 for a list of the items and the factor loadings. One item about intensively following the COVID‐19 news was omitted because it had factor loading of 0.25 or less on all the four dimensions.

**TABLE 1:** Baseline sociodemographic characteristics in 36 participants with bipolar disorder

	**No. (%) or median (P_25_–P_75_) or mean (SD) (*N* = 36)**
**Sociodemographic characteristics**:	
Male, sex	16 (44.4%)
Age; mean (SD)	36.7 (12.6)
Level of education:	
‐ Primary	2 (5.6%)
‐ Secondary	14 (38.9%)
‐ Higher	20 (55.6%)
Current smoker	12 (34.3%)
Alcohol use	
‐ None	13 (37.1)
‐ 1–4 units per month	15 (42.9)
‐ ≥ 4 units per week	2 (5.7)
‐ A history of alcohol use	4 (11.4)
Drug abuse	5 (14.3)
Marital status	
‐ No partner	20 (60.6%)
‐ With partner (not married)	4 (12.1%)
‐ Married	6 (18.2%)
‐ Divorced	3 (9.1%)
Children (yes)	12 (37.5%)
**Clinical characteristics**:	
Bipolar disorder type 1	11 (30.6%)
Age of onset; mean (SD)	
Age of onset first (hypo‐) mania	22.9 (7.2)
Age of onset first depression	20.4 (8.5)
Age of onset disease	18.9 (7.5)
Number of episodes:	
‐ No. of (hypo)manic episodes; median (P_25_–P_75_)	4 (2, 13)
‐ No. of depressive episodes; median (P_25_–P_75_)	7 (6, 14)
QIDS baseline; mean (SD)	11.2 (6.4)
YMRS baseline; mean (SD)	3.3 (3.8)
Medication use baseline:	
‐ Lithium	20 (57.1%)
‐ Anti‐epileptics	4 (12.5%)
‐ Anti‐psychotics	10 (28.6%)
‐ Benzodiazepines	5 (15.6%)
‐ Antidepressants	8 (25.0%)

In order to assess the changes in symptoms of depression, (hypo)mania, worry, perceived stress, and loneliness before and during the COVID‐19 pandemic, the marginal mean scores were estimated for each wave. At baseline, the sum scores of QIDS, YMRS, and PSS were also available. In addition to these questionnaires; ARSM, PSWQ, DeJong Q, and scores of the four symptom dimension scales were assessed during the COVID‐19 pandemic. Mixed models were used to compare marginal mean scores before and during the pandemic on symptom scales. All models were adjusted for age, gender, and the level of education.

Additionally, using linear regression analysis, we investigated whether age, gender, type of bipolar disorder and the four dimension scales of COVID‐19 questionnaire predicted the course of depression or (hypo)manic symptoms. For this analysis, all predictor variables were standardized for easier comparison of effect sizes among the different predictors. We tested for the interaction term of time * predictors, to explore whether some variables predicted for a stronger linear increase over time in mania (YMRS) or depressive symptomatology (QIDS, with time as a continuous variable). Finally, to compare the in‐ and out‐strengths of each of the 10 scale scores, we applied dynamic time ward (DTW) analyses of the time‐series of 20 BD patients with four or more assessments during their trajectories (Hebbrecht et al., 2020). The DTW distance between each pair of scale score was calculated (i.e., 40 distances per individual, for each of the 20 patients). In order to assess the direction of the effect, a asymmetric window type was used with the size of the time window of 1 (so only one assessment afterwards was taken into account). The descriptive analysis and EFA were done in SPSS version 22. We used packages in R (version 3.6.0) for linear regression, mixed models (package "lme4", version 1.1−21, and "emmeans", version 1.4.3.01), for DTW analyses (“dtw”, version 1.21‐3), and for figures (“forestplot”, version 1.9).

## 3 RESULTS

### 3.1 Sample characteristics

Basic demographic and clinical characteristics of the sample (*N* = 36) are summarized in Table 1.

The included subjects had a mean age of 36.7 (SD = 12.6) years and 44% were male. Two‐third of the participants were diagnosed with bipolar disorder type II (68.6) and had no partner (60.6%). At baseline, QIDS score was 11.2 (SD = 6.4), indicating moderate depressive symptoms and YMRS was 3.3 (SD = 3.8) indicating a low average severity of mania symptoms. Additionally, none of the participants had COVID‐19 before or during the time of the study, nor had any of their close relatives.

### 3.2 Changes in (hypo)manic, depressed, and stress‐related symptomatology before and during the COVID‐19 pandemic

**FIGURE 1:**
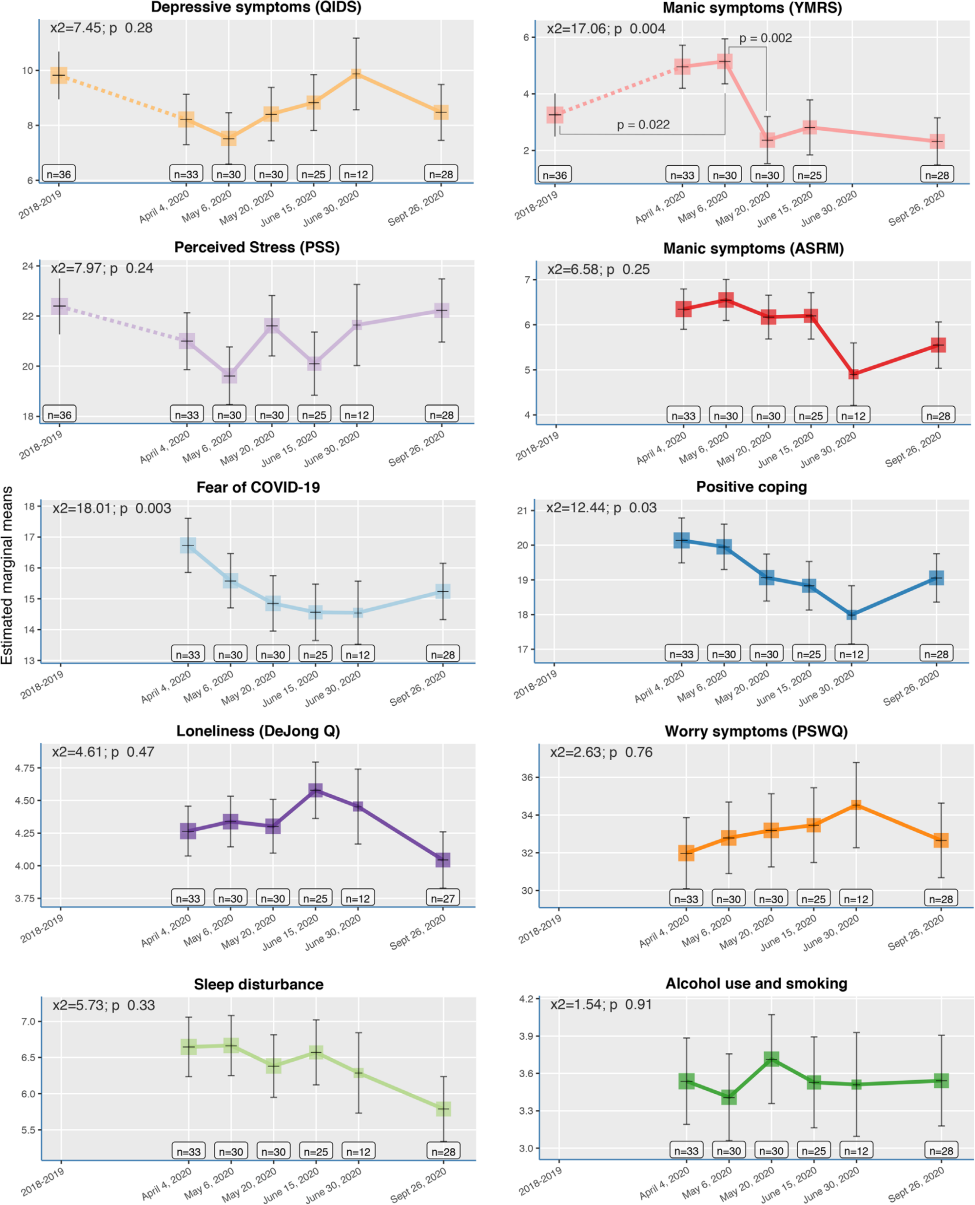
Trajectories of marginal means. Trajectories of marginal mean symptom severity scores before and during the COVID‐19 pandemic of symptoms of (hypo)mania (YMRS), depression (QIDS), and perceived stress (PSS). Trajectories of marginal mean scores of COVID‐19 related symptoms (fear of COVID, positive coping, sleep disturbances, alcohol use and smoking), loneliness (DeJong Q), worry (PSQQ), and self‐reported (hypo)mania on the ASRM during the pandemic. The number of included participants per wave are shown, and the size of each box is proportional to the number of subjects. Error bars represent standard errors. *p*‐Values by multilevel linear (mixed) models for the effects of time

Figure 1 depicts average marginal mean levels over time of the three symptom scores (i.e., QIDS, YMRS, and PSS) before and during the pandemic. Results of the mixed models analyses showed significant changes (X^2^ = 17.06; *p* = .004) in manic symptoms (YMRS) from baseline (prepandemic) to the COVID‐19 pandemic period. Compared to pre‐COVID‐19 levels, manic symptoms increased significantly during the first two time points (T2 and T3 between April and May), which coincides with the most strict lockdown measures in the Netherlands. Mania severity decreased significantly from T2 to T3 (end of May) and stayed stable from that time point onwards. Self‐reported manic symptoms (ASRM) were only measured during the COVID‐19 pandemic (no pre‐pandemic data available). Self‐reported (hypo)mania on the ASRM indicate an overall clinically significant (hypo)manic state (mean > 6), from April until June. No significant changes over time were found.

The depression symptoms (QIDS) showed a reverse trend, with symptoms decreasing at the beginning of the pandemic compared to baseline (prepandemic) and increasing back to baseline level during the summer, when lockdown measures were eased down. However, depressive symptom changes do not vary significantly over time, and overall mean symptom levels remain relatively mild both before and during the pandemic. No significant change from baseline to the pandemic was found for reported perceived stress among the bipolar patients.

### 3.3 Changes in COVID‐19‐related symptoms, loneliness, and worry during the pandemic

Results of the four dimension scales of COVID‐19‐specific questionnaires (see Figure 1) showed significant changes in fear of COVID‐19 (X^2^ = 18.01; *p* = .003) and positive coping (X^2^ = 12.44; *p* = .03) during the pandemic. In the beginning of the lockdown in April, the fear of COVID‐19 was the highest and it decreased significantly during the crisis. Positive coping showed the same trend, it was highest in the beginning of the crisis and it significantly decreased during the pandemic.

No significant mean changes over time were found for the COVID‐19 specific scales of sleep disturbances, alcohol use and smoking, and for worry, and loneliness. Worry levels were consistently high (mean ≥32) during the pandemic. We have repeated the analysis while removing T5 from the analyses, which resulted in similar significance levels for 9 out of the 10 outcomes. The trajectories for Fear of COVID‐19 and Manic symptoms were still statistically significant (*p* = .003 and *p* = .004, respectively), whereas the trajectory of Positive coping was no longer statistically significant (*p* = .11 instead of *p* = .03).

### 3.4 Predictors of mood symptoms

We then explored whether any of the seven factors (i.e., age, sex, BD type II vs. I, fear of COVID‐19, positive coping, sleep disturbance, and alcohol use and smoking) could predict for differential trajectories over time in (hypo)manic (YMRS) and depressive symptomatology (QIDS). Therefore, these potential interaction with time of these predictive factors were analyzed. Findings showed that none of these seven factors had significant predictive value for the course of (hypo)manic and depressive symptoms over time (see Figure S2). Finally, we explored which change in symptom scale score preceded other changes in symptom scale score in 20 participants with four or more COVID‐19 assessments (see Figure S3). We found that (hypo)manic and worry symptoms had the strongest out‐strengths, which persisted when we divided the BD group into two random subgroups of 10. This indicates that increases and decreases in (hypo)manic and worry symptoms tended to be followed by increases and decreases of other scales, rather than vice versa.

## 4 DISCUSSION

The current prospective study is among the first to investigate the impact of the COVID‐19 pandemic and its lockdown measures on recently diagnosed BD patients in an existing cohort. In the current study, we compared mania, depression, anxiety, and stress‐related symptom levels before the pandemic with levels during the pandemic using up to six follow‐up measurements in relatively young patients with bipolar disorder. We found that observer‐rated (hypo)mania symptoms increased significantly during the first 2 months of the pandemic compared to the (hypo)mania levels before the pandemic. Further, during the pandemic, fear of COVID‐19 and positive coping started off relatively high, but decreased significantly in the following months.

The initial increase of (hypo)manic symptoms during the pandemic compared to pre‐pandemic mania levels was rather mild, based on the clinician‐rated YMRS, although, the self‐reported scores on the ASRM during the pandemic did suggest clinically significant (hypo)manic symptomatology. Combining these findings, it seems that there was a meaningful increase in (hypo)manic symptoms among BD patients during the initial phase of the pandemic. One explanation for this increase could be the rather disruptive effect of the lockdown measures on the daily lives of the bipolar patients. Important cues for daily rhythms, such as going to work or study, bringing the kids to school, going to sport‐clubs or other hobbies disappeared during the lockdown when everyone was expected to live and work from home as much as possible. BD patients might be particularly vulnerable to such disruptions in daily rhythms, which have been related to the onset of new mood episodes (Alloy et al., 2015). Three recent case reports support associations between COVID‐19 pandemic and (hypo)manic symptoms. These case‐studies all describe cases of individuals who developed manic (psychotic) episodes during the COVID‐19 pandemic, both in people with no prior history of any psychiatric condition (Noone et al., 2020; Yin et al., 2020) and in patients who were already familiar with BD (Uvais, 2020). In all these cases, the stress and daily rhythm disruptions of lockdowns and/or quarantine were presumed to be a trigger for the (first) onset of manic episodes. The fact that a previous study (Orhan et al., 2020) among older patient with BD found a decrease in manic symptoms, while in our younger‐aged cohort we found an increase might be explained by the fact that younger people with BD are more vulnerable to life stressors than older adults. According to this inoculation‐hypothesis, older adults are better able to deal with life‐stressors because they simply have more experience with this (Knight et al., 2000).

Lastly, besides the fact that it is likely that the disruptive lockdown measures caused affective instability in BD patients in the current study, this association might be spurious since the start of the lockdown and the rise of the (hypo)manic symptoms coincide with the spring season. Spring and the increase of daylight have been repeatedly associated with increases in (hypo)manic symptomatology (Geoffroy et al., 2014) and therefore has to be mentioned as an alternative explanation.

Additionally, the disrupting effects for daily life of the pandemic, and the accessibility of mental health care during the lockdown could also contribute to increased instability. Although a recent study in the Netherlands showed that the availability of online treatment in many cases increased accessibility for patients, these methods also could lead to the missing of crucial information about a patient and rapid response to crises (Feijt et al., 2020).

In the current study, we observed a slight increase in depressive symptoms, although not significantly. Although cross‐sectional studies have suggested that an increase in depressive symptoms was associated with the start of the pandemic (e.g., Hao et al., 2020), results from prospective, repeated measures, studies showed that participants with mental illness had higher levels of depressive and anxiety symptoms, but these symptoms decreased in the subsequent weeks of lockdown (Fancourt et al., 2020; Pan et al., 2021). For bipolar patients specifically, cross‐sectional studies again indicated an increase in depressive symptomatology compared to comparison groups (Carta et al., 2021; Van Rheenen et al., 2020), but prospective studies showed no substantial increases in depressive symptomatology compared to prepandemic measures (Orhan et al., 2021; Yocum et al., 2021). Possible explanations are that most patients have previously dealt with stressful events and social isolation caused by their mental illness and therefore have learned how to cope with stressful situations, and that the lockdown measures induced some sense of relaxation as their world and habits became more in sync with the quarantined society.

During the pandemic we found that fear of COVID‐19 decreased significantly over time in BD patients. Presumably, with infection‐ and death rates decreasing, the fear of COVID‐19 was also wearing off. A comparable trend in fear of COVID‐19 during the course of the pandemic has been described in a previous study in the general population (Hetkamp et al., 2020). However, because we did not include a non‐psychiatric comparison group, it is impossible to state that the initial fear of COVID‐19 is related to having BD.

Although fear was increased during the initial months in the current study, we also found that positive coping was high. During the first months, the use of positive coping styles like staying active, feeling connected, and being socially connected to other people started rather high, but decreased over the following months. This trend could be caused by patients trying to make the best of it at the beginning of the pandemic, but failed to keep this positivity when time progressed. Previous studies in the general population only found weak associations between positive coping styles and well‐being (Dawson & Golijani‐Moghaddam, 2020; Zacher & Rudolph, 2020), which might lead to demotivation over time to maintain a positive attitude. Alternatively, the positive attitude in the current study seems to show a parallel course with the increase and decrease of (hypo)manic symptoms, so it could also be related to the energetic, positive, and often socially active (hypo)manic mood state. Either way, it seems that the current sample of patients with recent‐onset BD was able to use a positive coping style during the strictest lockdown measures, and this attitude decreased somewhat when lockdown measures were eased down. In addition, 55.6% of the participants were highly educated. Associations have been found between the level of education, emotional intelligence, resilient behaviors, and coping skills (Kristenson et al., 2004). Coping in the face of the COVID‐19 pandemic may have been moderated by the level of education, which should be studied in larger samples of BD patients.

The current study is among the first to repeatedly assess symptoms in the same BD patients both before and during the COVID‐19 pandemic. The sample contains recently diagnosed bipolar patients of a relatively young age, in the active stages of their lives (with study, work, and family circumstances) in which the impact of the lockdown is highly invasive, and therefore this is an important at‐risk group to study.

There are also some limitations to the current study. First, the sample size is rather small. Of the original 70 patients included in the BINCO study, only 36 participated in the current study. However, there were no differences between these in age, gender, education level, type of BD, and severity of depression or (hypo)manic symptoms among participants and non‐responders. Our findings were limited to a relatively small sample of recently diagnosed BD patients and may have been underpowered to detect more subtle trends in mental health. Nevertheless, at this point in time, few studies have investigated BD effects before and after COVID‐19.

Further, we did not gather data on some other variables that may explain some of the changes found over time (e.g., comorbid psychiatric disorders, vulnerability to seasonal variations, or non‐adherence to medication). Additionally, the use of self‐report measures might have let to response biases.

Another limitation is the lack of a healthy comparison group. As a consequence, we were not able to determine whether BD patients were more, less, or equally affected by the pandemic compared to healthy controls. A previous study among depressed and anxious patients showed that worry and loneliness symptoms were increased in the non‐psychiatric control group (Pan et al., 2021). It is likely that our BD patients were already higher in symptomatology compared to non‐psychiatric controls, and that these did not further increase during the pandemic. Moreover, although we had six repeated measurement points, no data were collected during summer break because of an anticipated lower response rate due to summer holidays of the participants. Additionally, we followed patients throughout the full first lock‐down period of six months, but given the long duration of the pandemic, longer follow‐up times might give a more complete view of the impact.

## 5 CONCLUSION

The most important finding of the current study is that there is a meaningful increase in (hypo)manic symptomatology in recently diagnosed bipolar disorder patients during the initial phases of the COVID‐19 pandemic compared to pre‐pandemic symptomatology. (Hypo)manic and worry symptomatology were the most influential with regard to all affective and COVID‐19 related scales. Therefore, it could be hypothesized that to limit the psychological and psychiatric impact of a stressful crisis like the COVID‐19 pandemic on BD patients, it would probably be effective to target worry and (hypo)manic symptoms in psychotherapeutic and pharmacological treatment sessions.

Since the increase in (hypo)manic symptomatology was rather mild, and no severe manic (psychotic) decompensations occurred in the current sample, these results could be interpreted as a sign of resilience and adaptability of this population, which has been proposed recently (Stefana et al., 2020). It is even more important that this resilience already seems to be present in this relatively young sample, that only recently started treatment, and therefore might be relatively unfamiliar with bipolar‐specific coping mechanisms to remain stable. Nevertheless, the increase in symptoms still means that BD patients need to be closely monitored (despite lockdown measures) during this pandemic, and future national and international crises as has been outlined by clinicians in the field (Hernandez‐Gomez et al., 2021).

## CONFLICT OF INTEREST

All authors declare no conflict of interest.

### PEER REVIEW

The peer review history for this article is available at https://publons.com/publon/10.1002/brb3.2326

## Supporting information

Supporting Information

## Data Availability

The data that support the findings of this study are available on request from the corresponding author. The data are not publicly available due to privacy or ethical restrictions.
