# Supplementary material for: Effects of the COVID‐19 pandemic in a preexisting longitudinal study of patients with recently diagnosed bipolar disorder: Indications for increases in manic symptoms
Source: Brain Behav. 2021 Sep 23;11(11):e2326. doi: 10.1002/brb3.2326 (PMC8613426; doi:10.1002/brb3.2326)
Supplement: Supplementary file 1 — Supporting Information [file BRB3-11-e2326-s001.pdf]

Supplementary Figure 1. Timeline of the study and the development of the COVID-19 pandemic in the Netherlands
Outline of the study measurements and COVID-19 lockdown measures during the study period. Bars indicate the development of COVID-19 pandemic in the Netherlands in terms of infection rates and number of death. The measurements and number of respondents are given below the timeline. T1 to T6 represents the 6 timepoint measurements during the study period.

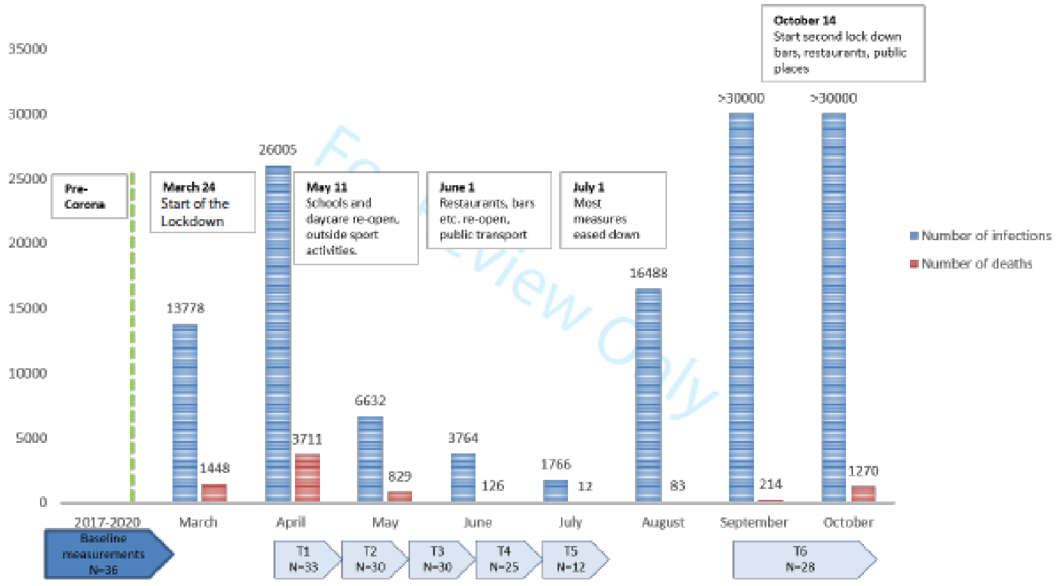

Supplementary Figure 2. Predictors for differential changes over time of symptoms trajectory. Predictors for differential changes over time of (hypo)manic (YMRS) and depressive (QIDS) symptoms trajectory. Data are standardized beta-coefficients of the interaction terms of time * predictor, from univariate mixed models (with time as a continuous variable).

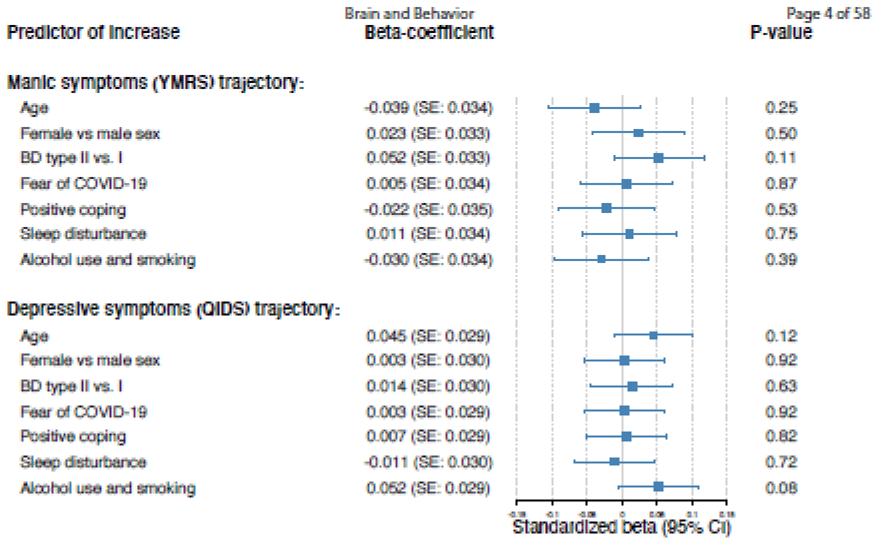

Supplementary Figure 3. In- and out-strength values of (DTW) analysis.
Standardized in- and out-strength values of the direct network plot based on dynamic time warp (DTW) analysis of the time series of 20 BD patients with 4 or more assessments over time (Hebbrecht et al. 2020). Changes that occur on scales with high outstrength values tend to precede (in time) changes in other scales, whereas scales with high instrength values tend to follow (in time) changes in other scales.

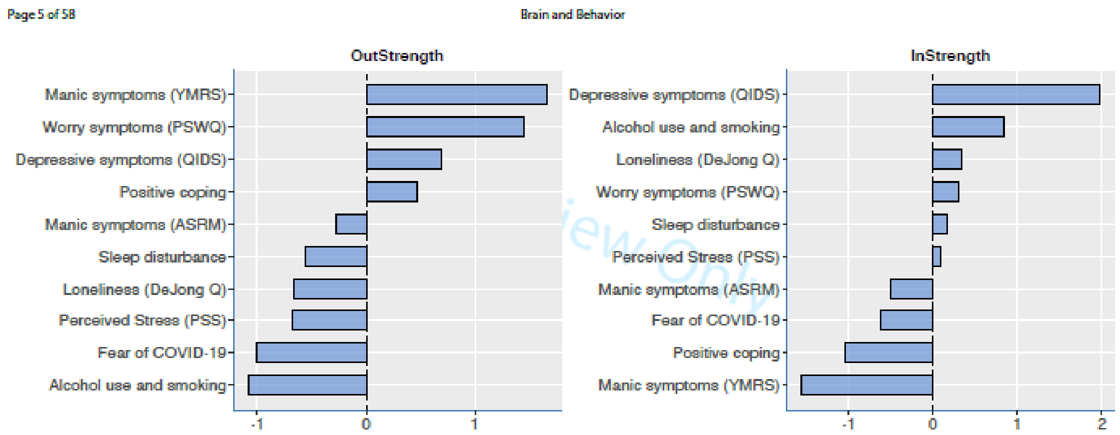

Supplementary Table 1: individual Covid-specific items and Exploratory Factor Analysis results (N = 33). The four dimensions are labelled as 'sleep disturbance', 'fear for COVID-19', 'positive coping' and 'alcohol use and smoking'.

**Item (response options: 1=totally disagree – 5=totally agree)**	**Factor loading 1**	**Factor loading 2**	**Factor loading 3**	**Factor loading 4**
Because of this period the quality of my sleep is worse	.70	.33	.14	.45
In this period I am having more nightmares	.46	.27	-.20	.31
In this period my daily structure (sleep hygiene, healthy food and sport) has got worsen.	.42	.21	-.10	.14
I fear to become infected with corona	.14	.72	.00	-.13
I fear that my loved ones to become infected with corona	-.02	.70	.48	-.24
Because of the threat of the virus I do not leave my home anymore	-.12	.56	-.30	-.19
This period makes me fearful	.37	.55	-.03	.14
Because of the threat of the virus I am anxious of getting close to other people	-.30	.95	.21	-.48
Because of the threat of the virus I am worried about the stability of my mood	.35	.49	-.25	.18
In this period I feel more connected to society	.43	-.04	.47	.07
It is no problem to enjoy myself while being at home more often	-.02	-.13	.75	-.16
I have confidence that the Netherlands will overcome this crisis	-.07	.05	.86	-.09
Despite the virus I stay active (household tasks, gardening, walking, sporting, yoga)	.02	.02	.62	.04
Despite the virus I actively maintain (via phone or online) contacts with friends	-.06	-.05	.53	-.03
Because of corona I have become more active (social media, helping others, more productive)	.40	.15	.56	.29
This period makes me smoke more	.02	-.01	.16	.83
This period makes me drink more alcohol (non-drinker: not applicable)	.11	0.22	-.03	.54
Dimension label	Sleep disturbance	Fear for COVID-19	Positive coping	Alcohol use and smoking
Cronbach's alpha	.60	.81	.79	.70
